# Genetic Targets of Hydrogen Sulfide in Ventilator-Induced Lung Injury – A Microarray Study

**DOI:** 10.1371/journal.pone.0102401

**Published:** 2014-07-15

**Authors:** Sashko Spassov, Dietmar Pfeifer, Karl Strosing, Stefan Ryter, Matthias Hummel, Simone Faller, Alexander Hoetzel

**Affiliations:** 1 Department of Anaesthesiology and Intensive Care Medicine, University Medical Center Freiburg, Freiburg, Germany; 2 Genomics Core Lab, Dept. Hematology, Oncology and Stem Cell Transplantation, University Medical Center Freiburg, Freiburg, Germany; 3 Joan and Sanford I. Weill Department of Medicine, New York-Presbyterian Hospital, Weill Cornell Medical College, New York, New York, United States of America; University of Louisville, United States of America

## Abstract

Recently, we have shown that inhalation of hydrogen sulfide (H_2_S) protects against ventilator-induced lung injury (VILI). In the present study, we aimed to determine the underlying molecular mechanisms of H_2_S-dependent lung protection by analyzing gene expression profiles in mice. C57BL/6 mice were subjected to spontaneous breathing or mechanical ventilation in the absence or presence of H_2_S (80 parts per million). Gene expression profiles were determined by microarray, sqRT-PCR and Western Blot analyses. The association of Atf3 in protection against VILI was confirmed with a Vivo-Morpholino knockout model. Mechanical ventilation caused a significant lung inflammation and damage that was prevented in the presence of H_2_S. Mechanical ventilation favoured the expression of genes involved in inflammation, leukocyte activation and chemotaxis. In contrast, ventilation with H_2_S activated genes involved in extracellular matrix remodelling, angiogenesis, inhibition of apoptosis, and inflammation. Amongst others, H_2_S administration induced Atf3, an anti-inflammatory and anti-apoptotic regulator. Morpholino mediated reduction of Atf3 resulted in elevated lung injury despite the presence of H_2_S. In conclusion, lung protection by H_2_S during mechanical ventilation is associated with down-regulation of genes related to oxidative stress and inflammation and up-regulation of anti-apoptotic and anti-inflammatory genes. Here we show that Atf3 is clearly involved in H_2_S mediated protection.

## Introduction

Mechanical ventilation can induce lung injury in the healthy lung or exacerbate pre-existing lung injury, both conditions which are referred to as ventilator-induced lung injury (VILI). Lung injury develops during mechanical ventilation as the result of cyclic alveolar stretch, leading to tissue disruption, release of inflammatory mediators, influx of inflammatory cells, and pulmonary edema [1]. VILI remains a major problem in critical care medicine [2], despite the implementation of low tidal volume ventilation that has reduced the rate of morbidity and mortality [3].

In a search for alternative therapeutic strategies, we recently found that inhalation of hydrogen sulfide (H_2_S) during mechanical ventilation prevents VILI in mice [4]. The protective properties of H_2_S have been increasingly investigated in various models of hemorrhagic shock, ischemia–reperfusion, oxidative stress, endotoxemia, and bacterial sepsis [5]. H_2_S, toxic at high concentrations, is an endogenously synthesized gaseous signal transducer that is involved in many biological processes including inflammation, apoptosis, vasodilatation, and the induction of suspended animation-like states in rodents [5]–[7]. Upon mechanical ventilation, gaseous H_2_S exerts anti-inflammatory effects by limiting cytokine release and neutrophil transmigration [4]. Likewise, application of H_2_S donors, such as sodium hydrosulfide (NaHS) and sodium sulfide (Na_2_S), exerts anti-inflammatory, antioxidant, and reducing effects that contribute to protection against VILI [8], [9].

Despite the increasing amount of data on the protective properties of H_2_S in VILI, the underlying molecular mechanisms remain elusive [9]. Therefore, we utilized a microarray approach for large scale analysis of target genes in order to elucidate the therapeutic effects of H_2_S in VILI. This is the first study to demonstrate the influence of supplemental H_2_S on gene expression in a model of VILI. In addition to describing the genes differentially regulated in VILI [10]–[16], the present study focused on newly identified H_2_S target genes within several functional groups, including anti-inflammatory and anti-apoptotic pathways, regulation of extracellular matrix (ECM) remodelling and angiogenesis.

## Materials and Methods

### Animals

All animal experiments were approved and carried out in accordance with the guidelines of the local Animal Care Commission (Ethics Committee University of Freiburg, Freiburg, Germany, permissions No. G-07/25 and No. G-12/73).

Male C57BL/6N mice (24–26 g body weight) were obtained from Charles River Laboratories (Sulzburg, Germany). After induction of anesthesia with 90 mg/kg ketamine and 1 mg/kg acepromazine intraperitoneally (i.p.), animals were placed on a heating pad, and insertion of an arterial line as well as a tracheal tube was performed as previously described [4], [17]. Mice were randomized into 4 groups with n = 5 per group. Two control groups were allowed to breathe spontaneously either synthetic air or synthetic air supplemented with 80 ppm H_2_S for 6 hours. Two groups were ventilated with synthetic air or synthetic air with 80 ppm H_2_S for 6 hours using a rodent ventilator (Voltekenterprises, Toronto, ON, Canada) set to a tidal volume of 12 ml/kg, a frequency of 80–90 breaths/minute, and a positive end-expiratory pressure (PEEP) of 2 cm H_2_O. At the end of experiments, mice were sacrificed and bronchoalveolar lavage fluid (BALF) was collected and analyzed for neutrophil cell count. The left lung lobe was prepared for histological analysis as described previously [4]. The remaining tissue samples were snap-frozen in liquid nitrogen and stored at −80°C for subsequent experiments.

### Histological Analysis

Cryosections (6 µm) were cut from the left lung lobe and subjected to hematoxylin and eosin (H&E) staining. At least five representative photographs were taken from each lung. In addition, five high-power fields were randomly assigned to each photograph. The degree of lung damage was determined in each power field using a modified VILI score: (1) thickness of the alveolar walls, (2) infiltration or aggregation of inflammatory cells, and (3) haemorrhage as described elsewhere [4], [18], [19]. The alveolar wall thickness was analyzed with Axiovision software (AxioVs4.0, Zeiss, Germany).

### RNA extraction and Microarray analysis

The inferior right lung lobe was homogenized in Trizol reagent (Invitrogen Corporation, Burlington, Canada). RNA was purified using the RNeasy Plus Mini Kit (Qiagen, Hilden, Germany) according to the manufacturer's instructions, and further processed with the Affymetrix GeneChip Whole Transcript Sense Target Labelling Assay as described by the manufacturer (Affymetrix UK Ltd, Mercury Park, UK). For details and microarray data processing see Materials and Methods S1.

Genes that showed statistical relevance after T-test (p≤0.005), false discovery rate (FDR) ≤10% and effect size ≥1.7 were further considered as target genes. The microarray dataset discussed in this publication have been deposited in NCBI's Gene Expression Omnibus [20] and are accessible through GEO Series accession number GSE58169.

### Gene enrichment analysis

To reveal dataset-specific gene ontology (GO) processes and their possible interactions, a gene enrichment analysis (GEA) was used (MetaCore software, http://www.genego.com/metacore.php). The input dataset included all differentially regulated genes meeting our restriction criteria. The most significant GO processes and GO process networks were depicted in a colour map. The colour map was created with the Multi Experiment Viewer software [21]. Colour intensity codes the annotation significance and the P value is presented as a log_10_ ratio.

### Semi-quantitative real-time PCR

cDNA samples were synthesized from equal amounts of RNA using random hexamer reverse primers and a Taqman Reverse Transcription kit (Applied Biosystems Inc, Foster City, USA). TaqMan PCR reactions were performed according to the manufacturer's instructions using TaqMan Universal PCR Master Mix and an ABI Prism 7000 device (Applied Biosystems). TaqMan Gene Expression Assays for Socs3 (Mm00545913_s1), Atf3 (Mm00476032_m1), Gadd45a (Mm00432802_m1), and GAPDH (TaqMan Rodent GAPDH Control Reagent) were purchased from Applied Biosystems. Gene expression in each sample was measured in triplicate. We used the comparative C_T_ (ΔΔC_T_) method to evaluate the expression profiles of the analyzed samples.

### Atf3 knockdown

Reduced Atf3 synthesis was achieved by administration of mRNA blocking Vivo-Morpholino oligonucleotides (Gene Tools, LLC Philomath, OR, USA). Mice (n = 4/group) received three intravenous (i.v.) injections of approximately 25 nmol per day of either Vivo-Morpholino oligonucleotides with a sequence 5′-TGAAGCATCATTTTGCTCCAGTC- 3′ complementary to the ribosome binding site of Atf3 mRNA (Atf3-Morpholino group) or non-binding Vivo-Morpholino standard control with a sequence 5′- CCTCTTACCTCAgTTACAATTTATA- 3′ (control-Morpholino group). One hour after the last Vivo-Morpholino instillation, mice were subjected to ventilation in the presence of 80 ppm H_2_S. Subsequent sample collection and data analysis were performed as described above. As controls, mice were either left breathing synthetic air spontaneously (air control group) or were ventilated with synthetic air for 6 hour (air ventilated group) as described above. Both control groups received equivalent i.v. administration of vehicle (phosphate buffer saline).

### Immunoblotting

The right upper lung lobe was homogenized in 30 mM Tris base solution with complete protease inhibitors (Roche Diagnostics, Germany). Equal amounts of protein samples were separated in 15% SDS-PAGE, blotted onto a polyvinylidene fluoride membrane and immunostained with the following antibodies: Socs3, Atf3 and GADD45a (Abcam, Cambridge, UK). The protein bands were visualized using CDP-star reagent (Applied Biosystems). The chemiluminescence signal was detected by exposure of the membrane to radiographic films (GE Healthcare, Freiburg, Germany). To control protein loading, the membranes were re-stained with an antibody against β-tubulin (Cell Signalling, Beverly, USA). Densitometry analysis was performed with Image J software (Wayne Rasband NIH, USA, http://rsb.info.nih.gov/ij).

### Statistical Analysis

The four analyzed groups included n = 5/group for the microarray and n = 4/group for the Atf3-Morpholino study. All microarray and sqRT-PCR data represent medians, and densitometric analyses of Western blots represent means, ±SEM as indicated. Statistical analyses were performed using one-way ANOVA for multiple-group comparison followed by the Student-Newman-Keuls post hoc test (Sigmastat statistical software; Systat, Inc., Germany). *P*<0.05 was considered significant.

## Results

### Hydrogen sulfide protects against VILI via anti-inflammatory effects

**Figure 1:**
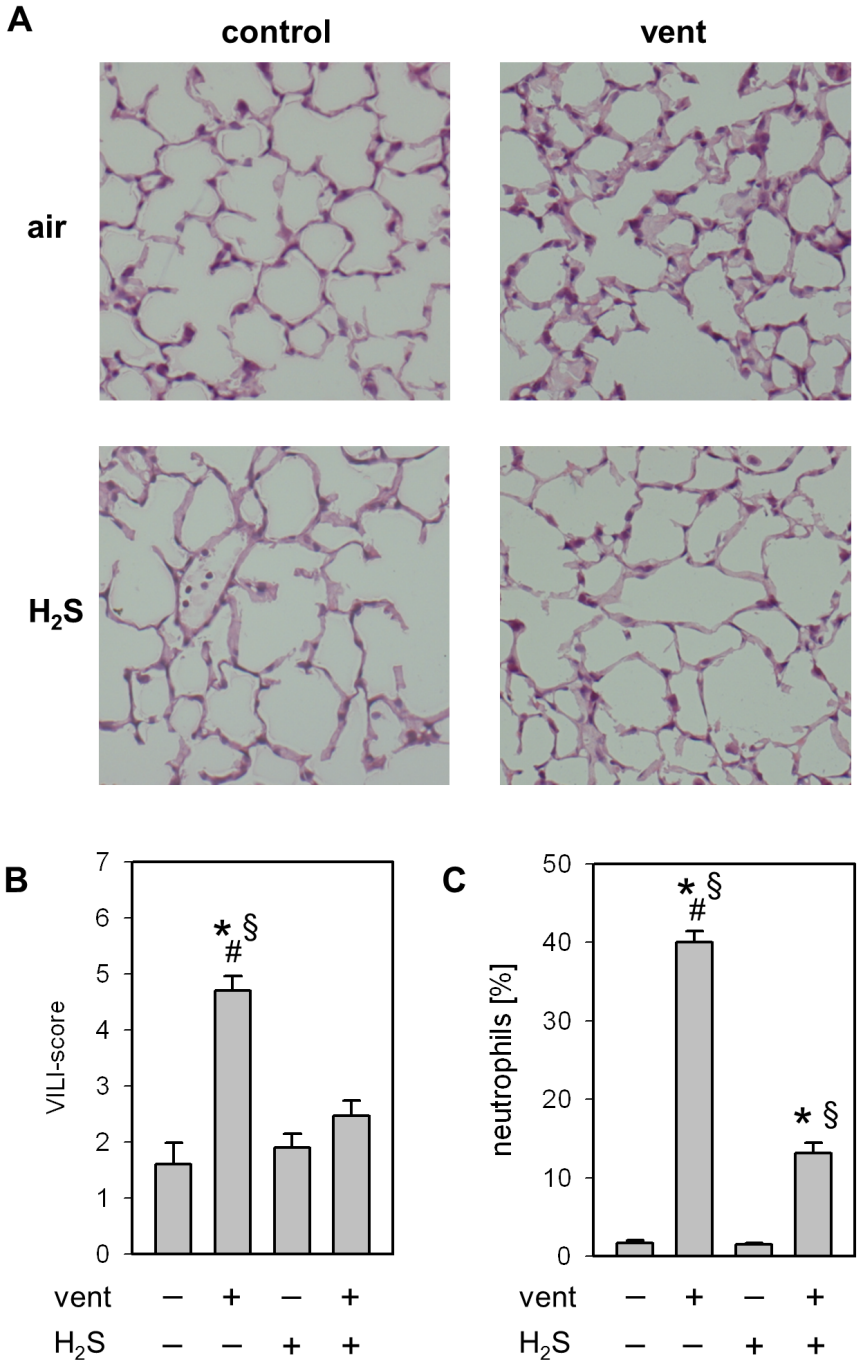
Effect of ventilation and hydrogen sulfide (H_2_S) treatment on lung injury. (A) Representative pictures of H&E stained lung tissue from animals mechanically ventilated (vent)(12 ml/kg, 6 h) in the absence or presence of supplemental H_2_S (80 ppm) and their corresponding non-ventilated controls. (B) Ventilator-induced lung injury (VILI) score was measured from the histology samples and (C) the relative amount of neutrophils was determined by cytospin analysis of BALF. Data represent means ±SEM for n = 5/group. Analysis of variance (Student–Newman–Keuls post hoc test), *P<0.05 vs. control; ^#^P<0.05 vs. H_2_S control; ^§^P<0.05 vs. H_2_S-ventilated group.

Lung histology and applied VILI score were comparable in animals that were allowed to spontaneously breathe synthetic air, or air with 80 ppm H_2_S (fig. 1 A+B). Animals ventilated with synthetic air showed significant thickening of alveolar walls, increased inflammatory cell infiltrates as well as a higher VILI-Score. In contrast, administration of 80 ppm H_2_S during ventilation abolished histological signs of lung injury. Thus, the VILI score of animals ventilated in the presence of H_2_S was comparable to control animals.

With respect to the inflammatory response to mechanical ventilation, the percentage of neutrophil cells in the BALF remained below 2% in spontaneously breathing control groups, irrespective of H_2_S application (fig. 1 C). Ventilation with air increased the neutrophil fraction to 40%, an effect that was abolished in the presence of H_2_S.

### Effect of mechanical ventilation and hydrogen sulfide on gene expression profiles

**Figure 2:**
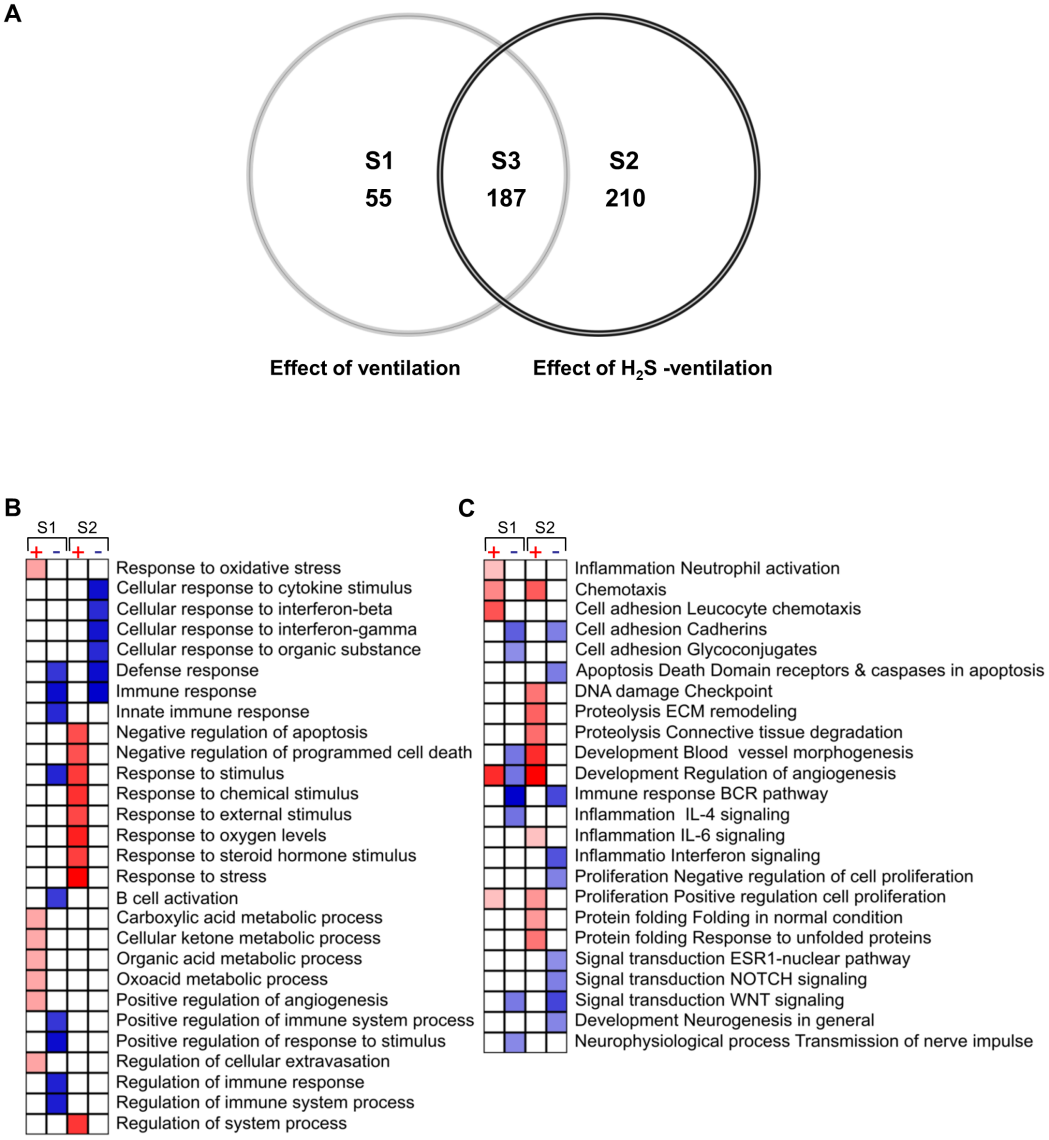
Venn diagram (A) showing the intersections between the lists of differentially regulated genes in response to 6 h mechanical ventilation (effect of ventilation, S1), mechanical ventilation with supplemented H_2_S (effect of H_2_S ventilation, S2) and, genes found differentially regulated in both groups (S3). The lists of effect-specific and intersection genes are shown in supplementary tables S1 to S3. Only genes with statistical relevance after unpaired Bayer T-test p≤0.005, false discovery rate (FDR) ≤10% and effect size ≥1.7 were included in the diagram (created using Genedata Analyst 2.2.6b software). Gene enrichment analysis (GEA) (B+C) of significant genes induced during mechanical ventilation (S1) and mechanical ventilation with H_2_S (S2). Effect size was transformed to log2 ratio prior GEA performed with the MetaCore software. Only significant events, p<0.02, FDR<15% and including minimum of 5 genes GO processes (B) and GO processes networks (C) are shown. Shades of red (up-regulated) and blue (down-regulated) coded the degree of significance of the corresponding annotation.

Next, we analyzed the gene expression response to spontaneous breathing and mechanical ventilation in the absence or presence of H_2_S (80 ppm). Spontaneous breathing of H_2_S minimally affected the gene expression profile as compared to breathing air alone. The expression of 16 genes was altered in response to H_2_S inhalation (*data not shown*). Mechanical ventilation substantially altered the gene expression pattern relative to controls. As depicted in the Venn diagram (fig. 2 A), 452 genes were differentially expressed. All genes were distributed among three groups: (1) 55 genes were specifically regulated by ventilation in the absence of H_2_S (fig. 2, table S1). (2) 210 genes were specifically regulated by ventilation in the presence of H_2_S (fig. 2, table S2). (3) In the intersection of these groups, 187 genes were regulated by ventilation irrespective of the presence or absence of H_2_S (fig. 2, table S3). None of the genes found to be differentially expressed in both ventilated groups were counter-regulated.

In the mechanical ventilation group, we detected a number of differentially regulated genes that were previously reported [10]–[14], [16]. Among these, we found genes involved in regulation of inflammation, stress response, tissue remodeling, transcription factors, molecular transport, kinases and phosphatases. These genes were up- or down-regulated in response to mechanical ventilation independently of H_2_S application (see tables S1, S2, S3).

### Effect of mechanical ventilation and hydrogen sulfide on gene ontology processes and networks

As described above, spontaneous breathing of H_2_S altered a very limited number of genes, too small for grouping and gene enrichment analysis (GEA). Therefore, we focused on analysis of the two ventilated groups. GEA showed that most of the gene ontology (GO) processes and GO process networks were specific for either the air–ventilated or the H_2_S–ventilated group. Mechanical ventilation favoured expression of genes involved in GO processes such as oxidative stress and metabolic processes (fig. 2 B). In contrast, ventilation with H_2_S activated processes involved in negative regulation of cell death and apoptosis, and response to environmental stimuli, (*e.g.*, chemical, oxygen levels, steroid hormones and stress).

Analysis of the GO process networks (fig. 2 C) revealed further physiological events and pathway activation in response to mechanical ventilation with or without H_2_S. For example, inflammation induced by ventilation with air appeared to be associated with neutrophil cell activation, enhanced leukocyte adhesion, and chemotaxis. Furthermore, ventilation with H_2_S promoted remodeling of the extracellular matrix (ECM) and connective tissue degradation as compared to ventilation alone. Finally, mechanical ventilation with air induced both up- and down-regulation of processes involved in development and control of angiogenesis, whereas ventilation with H_2_S contributed only to activation of angiogenesis control and blood vessel development.

### Effect of mechanical ventilation and hydrogen sulfide on Socs3, Atf3, and Gadd45a

**Figure 3:**
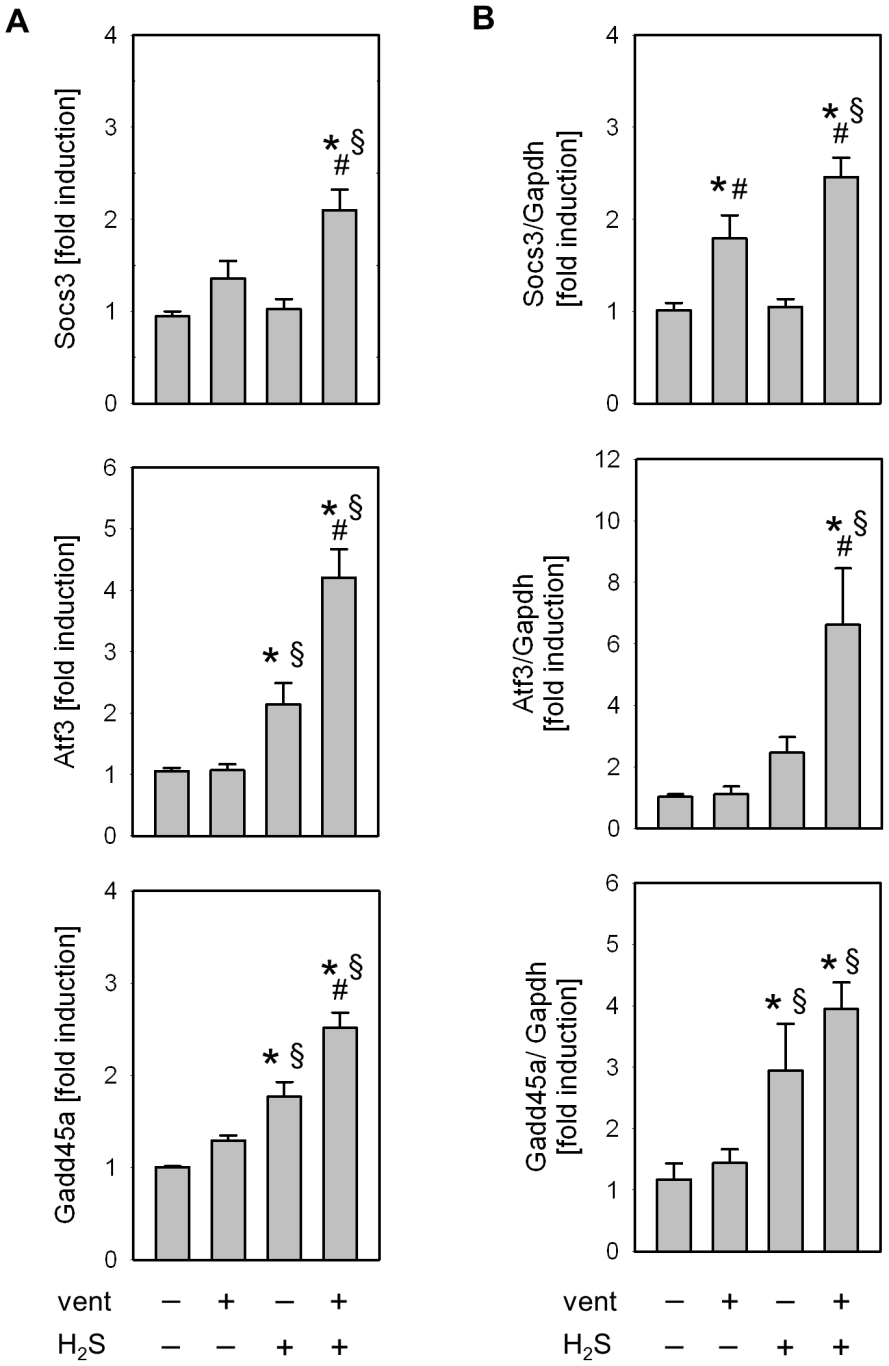
Verification of the microarray expression profiles of Socs3, Atf3 and Gadd45a by semi-quantitative RT-PCR. (A) Microarray expression levels of the control group (spontaneously air breathing animals) were used to calculate the effect of mechanical ventilation, spontaneous inhalation of H_2_S, and ventilation in the presence of H_2_S. (B) RT-PCR expression values for each gene were normalized to GAPDH. Data represent median of fold change ±SEM for n = 5/group. Analysis of variance (Student–Newman–Keuls post hoc test), *P<0.05 vs. control; ^#^P<0.05 vs. H_2_S control; ^§^P<0.05 vs. air-ventilated group.

**Figure 4:**
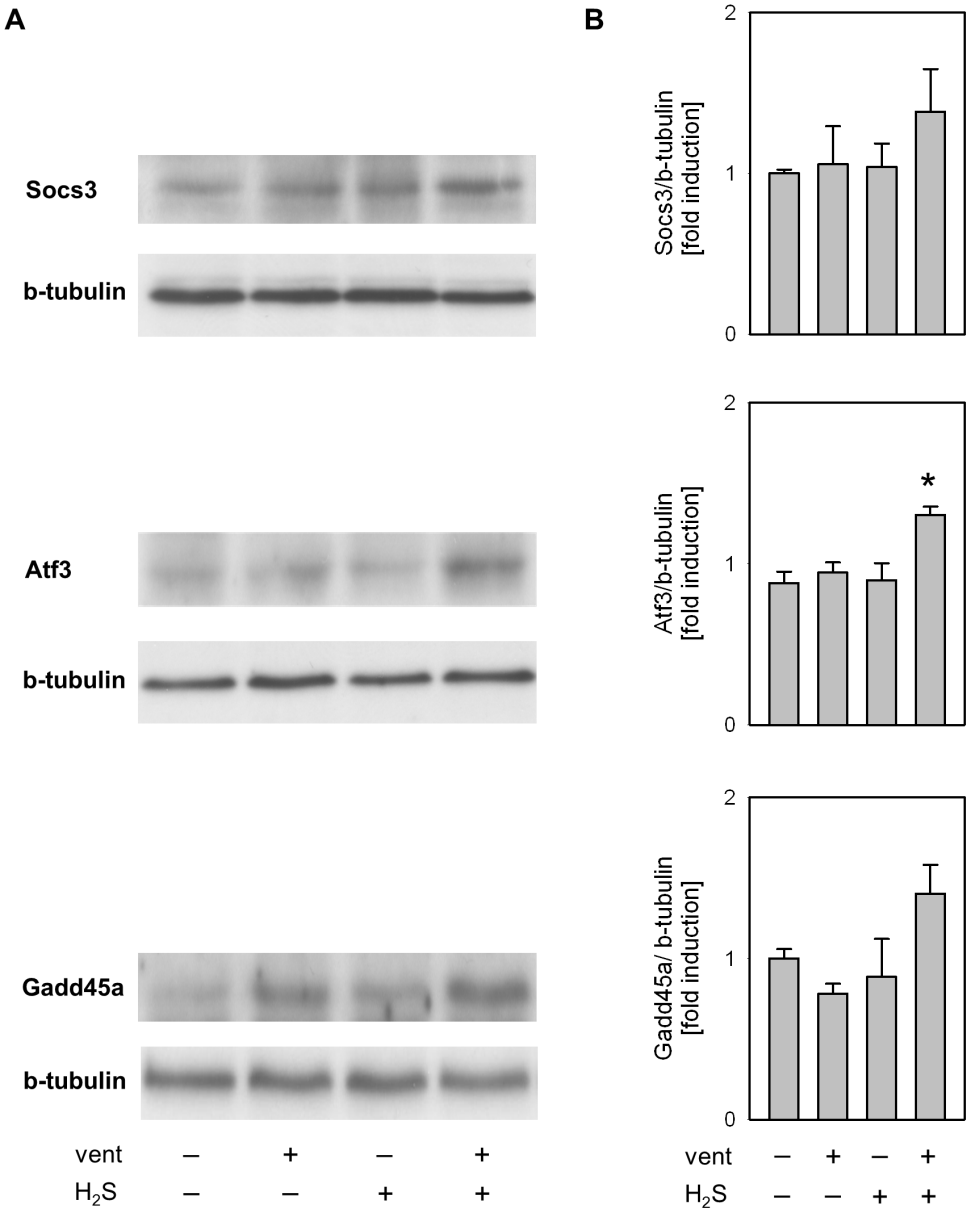
Western blot analysis of lung tissue homogenates showing increase of Socs3, Atf3 and Gadd45a protein synthesis in response to 6_2_S. (A) Representative Western blots for Socs3, Atf3, Gadd45a and β-Tubulin, and (B) corresponding densitometric analysis. All samples were normalized to β-tubulin and expressed as fold induction. Data represent mean ±SEM for n = 5/group. Analysis of variance (Student–Newman–Keuls post hoc test), *P<0.05 vs. control.

To validate the gene expression profiles from the microarray experiments, we performed sqRT-PCR and Western blot analyses on three genes induced during ventilation in the presence of H2S that have previously been shown to contribute to protection against VILI [22]–[27]: Socs3 (suppressor of cytokine signalling 3), Atf3 (activating transcription factor 3), and Gadd45a (growth arrest and DNA-damage-inducible 45 alpha). Gene expression profiles obtained from microarray and sqRT-PCR analyses (fig. 3 A+B) showed similar expression patterns for these genes. With respect to protein expression, Socs3, Atf3, and Gadd4a protein were further up-regulated in response to supplemental H_2_S inhalation as compared to ventilation alone (fig. 4). Thus, the expression patterns of these proteins were comparable to the mRNA results.

### Effect of Atf3 knockdown on VILI

**Figure 5:**
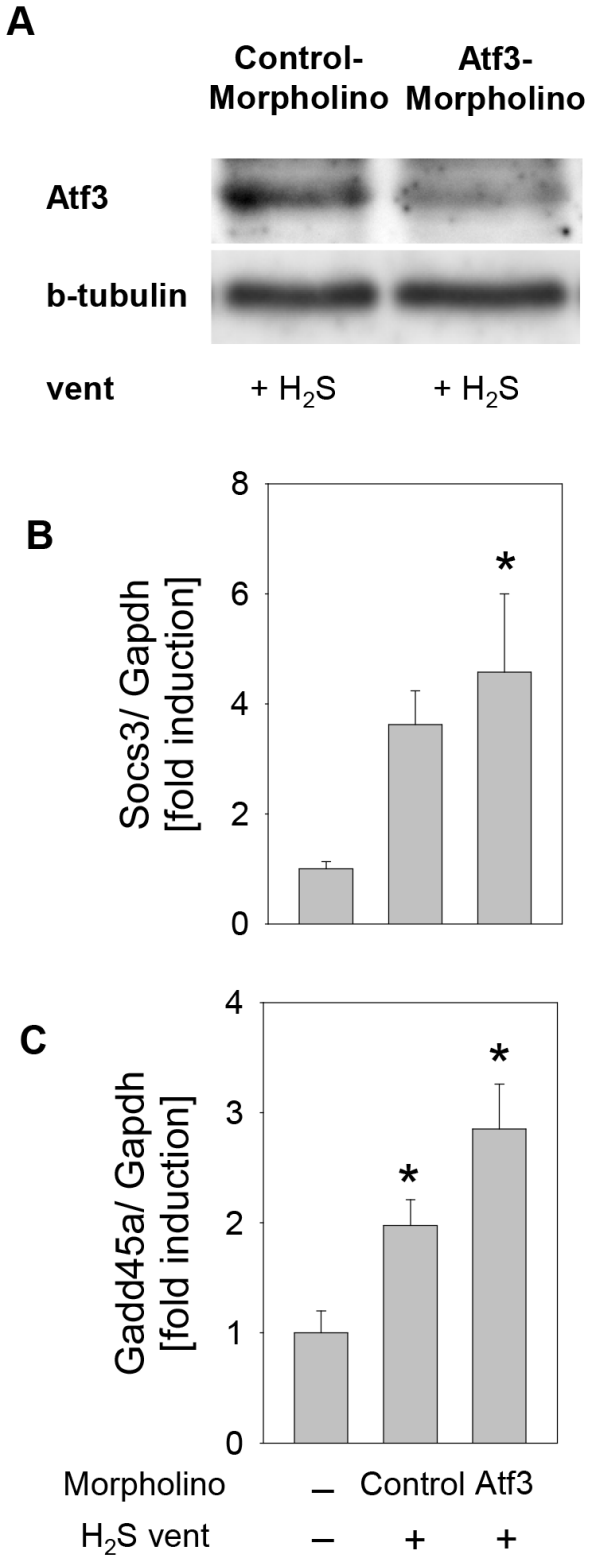
Effect of Atf3-Morpholino on Atf3 protein synthesis, Socs3 and Gadd54a gene expression. (A) Representative Western blot showing reduced Atf3 synthesis in mice treated with Atf3-Morpholino and subsequently subjected to mechanical ventilation in the presence of 80 ppm H_2_S. (B) and (C) RT-PCR expression values for Socs3 and Gadd45a normalised to GAPDH. Data represent median of fold change ±SEM for n = 4/group. Analysis of variance (Student–Newman–Keuls post hoc test), *P<0.05 vs. control.

Treatment of H_2_S-ventilated mice with Vivo-Morpholino oligonucleotides complementary to the ribosome binding site of Atf3 mRNA resulted in reduced Atf3 protein synthesis in lung tissue compared to the control-Morpholino treated group (fig. 5 A). Interestingly, ventilation with supplementary H_2_S resulted in slightly elevated Socs3 and Gadd45a gene expression levels in Atf3-Morpholino mice compared to the control-Morpholino mice (fig. 5 B+C).

**Figure 6:**
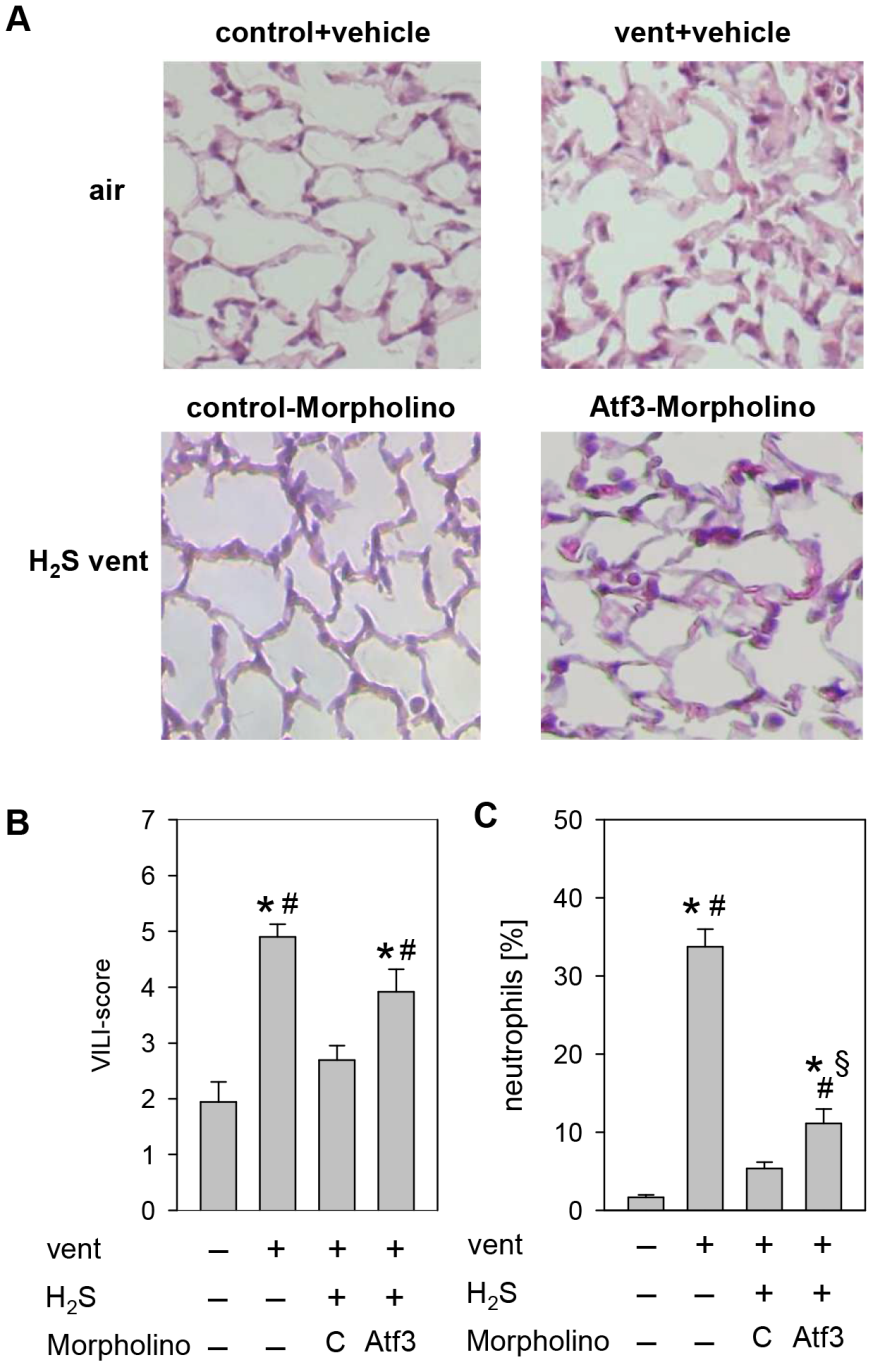
Effect of reduced Atf3 protein synthesis on VILI. (A) Representative pictures of H&E stained lung tissue from control animals and animals mechanically ventilated with synthetic air in the presence or absence of H_2_S. Control-Morpholino or Atf3-Morpholino treated mice were ventilated with supplemented H_2_S. (B) Ventilator-induced lung injury (VILI) score was measured from the histology samples and (C) the relative amount of neutrophils was determined by cytospin analysis of BALF. Data represent means ±SEM for n = 4/group. Analysis of variance (Student–Newman–Keuls post hoc test), *P<0.05 vs. control; ^#^P<0.05 vs. H_2_S control-Morpfolino; ^§^P<0.05 vs. air-ventilated group.

As described above, in this series, lung histology showed that ventilation induced lung injury that was prevented in the presence of H_2_S (fig. 6 A). These findings were not affected by the application of control-Morpholino. However, administration of Atf3-Morpholino in the presence of H_2_S clearly led to the development of VILI, demonstrated by thickened alveolar walls, increased inflammatory cell infiltrates, and occurrence of sporadic blood vessel leakages. Furthermore, in comparison to the two H_2_S-ventilated groups, quantification of the histopathological changes revealed a significant increase of the VILI score in the Atf3-Morpholino group as compared to the control-Morpholino group (fig. 6 B). Most interestingly, the prevention of neutrophil infiltration by H_2_S was still existent in the Atf3-Morpholino group, indicating that the organ-protective effect, and in part the anti-inflammatory effect of H_2_S, depended on Atf3 (fig. 6 C).

## Discussion

To perform a large scale screening of H_2_S-regulated genes that contribute to lung protection, we used a well established and clinically relevant VILI model [4], [17], [18], [28]. Histology, VILI scores, and inflammatory cell infiltrate counts showed that mechanical ventilation with a tidal volume of 12 ml/kg for 6 hours resulted in moderate lung injury in mice which was in turn prevented by the administration of 80 ppm H_2_S. These results are consistent with previous studies from our group and others demonstrating the protective capacity of H_2_S against VILI [4], [8], [9]. We preferred the use of spontaneous breathing control animals instead of mice ventilated with lung protective settings, *i.e.*, low tidal volume. First, in a preliminary study, we approved that mice subjected to mechanical ventilation with a protective setting (7 ml/kg tidal volume, 6 h) do not develop any signs of lung injury and were comparable to spontaneously breathing animals (data not shown). Second, we aimed to identify the overall gene expression changes as a result of mechanical ventilation with and without supplemented H_2_S, and third, we sought to observe exclusively H_2_S mediated gene regulation in control animals excluding ventilator effects.

Our findings led us to examine which genes and gene groups were affected by inhalation of H_2_S with or without mechanical ventilation. More than 452 genes were differentially regulated by mechanical ventilation and/or further modulated by ventilation with H_2_S. Virtually any of these genes could be responsible for the observed H_2_S-mediated protection. Therefore, the definition of gene groups and networks is essential. Gene enrichment analysis (GEA) revealed the differential regulation of key physiological processes (*e.g.*, oxidative stress, inflammation and ECM remodelling), which could contribute to injury or mediate lung protection in the absence or presence of H_2_S, respectively.

GEA showed an abundance of genes involved in inflammation, leukocyte activation, chemotaxis, and oxidative stress in lung samples of air-ventilated mice. Activation of macrophages and neutrophil cells, and redox imbalances in the lung are closely related to VILI [29], [30]. On the contrary, H_2_S ventilation in our VILI model prevented the onset of these processes. Furthermore, ventilation with supplemental H_2_S resulted in a particular gene expression profile that favours negative regulation of apoptosis and inflammation. Our study, and previous findings [4], [8], [9] confirm the anti-inflammatory and anti-apoptotic effects of H_2_S in mice models of VILI [5], [7]. Furthermore, the GEA revealed enrichment of genes involved in ECM remodelling. In our model, mechanical ventilation triggered the up-regulation of matrix metalloproteinase-8 (MMP-8), a protein that affects ECM remodelling. Upon activation, MMP-8 is involved in collagen I, II and III degradation. This protease can be expressed by a wide variety of cell types and plays an important regulatory role in both acute and chronic inflammation [31], wound healing [32] and cardiovascular diseases [33], [34]. MMP-8 deficient mice were shown to be sensitized to VILI [16]. In addition, ventilation with supplemental H_2_S resulted in the transcriptional up-regulation of Serpina3. Serpina3 is regarded as an acute-phase inflammatory protein that can protect tissues against proteolytic enzymes and promote repair in inflamed tissue [35]. Recent animal experiments provide evidence that H_2_S-induced ECM remodelling (*via* differential regulation of MMPs and their inhibitors) serves as an underlying mechanism for epithelial healing [36], or recovery in injury-induced vascular remodelling [37]. With respect to our study, pilot experiments showed that a late onset of H_2_S and short duration of application does not prevent lung injury. However, late onset of H_2_S inhalation but longer duration, *e.g.*, 5 hours air-ventilation followed by 5 hours H_2_S-ventilation still clearly reduces lung injury (data not shown). These preliminary results in orchestration with the microarray findings support the notion that H_2_S affects ECM, and thus, might contribute to the observed protection against VILI.

Application of H_2_S can regulate protective genes in the presence of injurious ventilation. This assumption is supported by our observation that several genes induced by H_2_S ventilation, (*i.e.*, Socs3, Atf3 and Gadd45a), were shown to play a significant role in the protection against VILI. Therefore, we addressed further evaluation of these three genes.

Socs3 is a cytokine-inducible negative regulator of cytokine signalling with established anti-inflammatory and anti-apoptotic effects [38], [39]. Socs3 was shown to contribute to lung protection after IL-22 challenge in a model of VILI [24]. Atf3 is critical for the prevention of acute inflammatory syndromes, including VILI, by limiting pro-inflammatory cytokine expression and may control the balance between proliferative and apoptotic signals [22], [40]. Gadd45a seems to be involved in microvascular barrier maintenance in the lung and may play a significant role in clinical predisposition to VILI [25], [41].

Studies in knockout mice have uncovered the significance of Atf3 and Gadd45a in VILI [22], [25]. Both knockout models have shown that lack of functional protein increased the severity of lung injury upon mechanical ventilation. Importantly, our microarray data demonstrated up-regulation of Atf3 and Gadd45a even in lung tissue of mice spontaneously breathing H_2_S. The ability of H_2_S to induce Atf3 is consistent with a previous study [36] and supports the notion that Atf3 may play an important role in H_2_S-mediated lung protection. As our results show, Atf3-Morpholino mediated down-regulation of Atf3 protein synthesis during mechanical ventilation inhibited the organ protective effects of H_2_S in VILI. It is important to note that the observed anti-inflammatory effect of H_2_S (*i.e.*, decreased neutrophil cell accumulation) appears to be only partly dependent on the Atf3 pathway. The slight induction of Socs3 and Gadd45a gene expression levels in the case of reduced Atf3 shows that these genes do not share the downstream pathway of Atf3 and rather suggest the possibility of complex feedback and/or compensatory mechanisms. Thus, additional unidentified signalling molecules could be involved in H_2_S-Atf3 mediated lung protection. This hypothesis is supported by the fact that the presented microarray findings indicate several regulatory processes upon H_2_S administration, together orchestrating H_2_S mediated organ protection.

## Summary

In this study we provide evidence on potential mechanisms involved in H_2_S mediated protection against VILI. H_2_S down-regulates genes that are involved in oxidative stress and pro-inflammatory cell responses. H_2_S regulates ECM remodelling, a mechanism which may contribute to H_2_S-mediated lung protection. In addition, H_2_S inhalation activates anti-apoptotic and anti-inflammatory genes, and genes controlling the vascular permeability. The functional relevance of Atf3 underscores the potential of H_2_S to limit lung injury.

## Supporting Information

Table S1 **Genes specifically regulated by ventilation in the absence of H_2_S.**
(XLS)

Table S2 **Genes specifically regulated by ventilation in the presence of H_2_S.**
(XLS)

Table S3 **Genes regulated by ventilation irrespective of the presence or absence of H_2_S.**
(XLS)

Materials and Methods S1 **Supplementary materials and methods.**
(DOCX)
