# Supplementary material for: Genetic Targets of Hydrogen Sulfide in Ventilator-Induced Lung Injury – A Microarray Study
Source: PLoS One. 2014 Jul 15;9(7):e102401. doi: 10.1371/journal.pone.0102401 (PMC4099342; doi:10.1371/journal.pone.0102401)
Supplement: Materials and Methods S1 — Supplementary materials and methods. (DOCX) [file pone.0102401.s004.docx]

## RNA extraction and Microarray analysis

All RNA samples showed a 260/280 ratio between 1.9 and 2.1 and a 260/230 ratio above 1.9 as measured with a Nanodrop ND-1000 device (Thermo Scientific, Wilmington, DE, USA). RNA integrity was analyzed by capillary electrophoresis using a 2100 Bioanalyzer (Agilent Technologies, Santa Clara, CA, USA). RNA samples with a RNA integrity number of greater than 9.0 were further processed with the Affymetrix GeneChip Whole Transcript Sense Target Labelling Assay as described by the manufacturer (Affymetrix UK Ltd, Mercury Park, UK).

Briefly, 500 ng of total RNA were reverse transcribed with (N6)-random primers coupled to a T7 RNA Polymerase promoter sequence. After second-strand synthesis, the double-stranded cDNAs were amplified overnight by *in-vitro*-transcription using T7 RNA Polymerase. The cRNA was purified and used as a template for a second cycle of reverse transcription with (N6) random primers. Here, dUTP was incorporated into the cDNA. After hydrolysis of the cRNA with RNAse H, the cDNA was purified and subsequently fragmented using the enzymes Uracil-DNA Glycosylase (UDG) and human apurinic/apyrimidinic Endonuclease 1 (APE1). The cDNA fragments were then labeled at their 3'-end with biotin using Terminal Deoxynucleotidyl Transferase (TdT). Labeled fragments were hybridized to the arrays for 16 h at 45°C with 60 rpm in an Affymetrix Hybridization Oven 640. After washing and staining, the arrays were scanned with the Affymetrix GeneChip Scanner 3000 7G. CEL-files were produced from the raw data with Affymetrix GeneChip Command Console Software Version 3.0.

Genedata Expressionist software (Genedata AG, Basel, Switzerland) was used for further data analysis. CEL files were imported into the Refiner (Version 6.1) module of Expressionist. GC background subtraction was performed using anti-genomic background probes. Subsequently, quantile normalization and probe summarization was performed using the Bioconductor RMA condensing algorithm as implemented in Refiner (Irizarry et. al., Biostatistics 2003). To identify differentially expressed genes between the groups, the unpaired Bayes T-test (CyberT) (Baldi and Long, Bioinformatics 2001) with the Bayes Confidence Estimate Value set to 15 and a window size of 101 genes as well as 100% valid values in each group was used with the Analyst module (version 2.2.6b). To control the false discovery rate (FDR), the Benjamini-Hochberg q-value was calculated (Benjamini and Hochberg, J Royal Stat Soc 1995). We then used the "Effect size" activity of Analyst to calculate the paired Effect size score between the experimental groups. Only genes from the categories "main" and "unmapped" (see Affymetrix transcript annotation NA32) were included in the analysis.
